# Management of Osteoporotic and Neuropathic Ankle Fractures in the Elderly

**DOI:** 10.1007/s13670-017-0196-y

**Published:** 2017-02-03

**Authors:** P Hoogervorst, CJA Van Bergen, MPJ Van den Bekerom

**Affiliations:** 1 Department of Orthopaedic Surgery, Onze Lieve Vrouwe Gasthuis, Oosterpark 9, 1091 AC Amsterdam, the Netherlands; 2 Department of Orthopaedic Surgery, Spaarne Gasthuis, Spaarnepoort 1, 2134 TM Hoofddorp, the Netherlands

**Keywords:** Ankle fracture, Elderly, Osteoporosis, Neuropathy, Multidisciplinary

## Abstract

**Purpose of Review:**

Treatment of osteoporotic and neuropathic ankle fractures in the elderly is challenging. The purpose of this paper is to review recent publications on this topic and to identify the optimal treatment for these fractures.

**Recent Findings:**

Treatment consists of a variety of conservative or operative options all with advantages and disadvantages as described in this review. Little research has been published that specifically focuses on elderly patients with ankle fractures. Operative treatment has a high complication rate. Multiple comorbidities are predictors for complications.

**Summary:**

An optimal treatment could not be distilled but based on the available literature, a general treatment algorithm is proposed. Since the elderly typically are accompanied by multiple comorbidities as well as impaired mobility, the physician should focus not only on treating the fractured ankle but also on the patient as a whole. Further research on this specific topic is needed.

## Introduction

Ankle fractures are common fractures with an incidence of 122–187 in. 100,000 persons a year [1, 2]. It is the third most common fracture in the elderly [2]. There is a bimodal distribution of those at risk for these types of fractures [2]: young, active men suffering from a high-impact trauma and older women with low-energy trauma. The latter is thought to be caused by impaired bone mineralization and obesity [3–5]. As the general life expectancy increases, so does the incidence of osteoporotic, neuropathic, and insufficiency ankle fractures.

Because adults ≥65 years comprise an increasing proportion of the population worldwide, the impact of these types of fractures on the health-care systems and societies will become larger in time. The aim of the present review was to identify the best current practice concerning osteoporotic and neuropathic ankle fractures, as described in recent available publications.

### Treatment Goals

The goal of treatment of the fractured ankle in the younger population is to obtain a stable and congruent tibiotalar joint in order to prevent posttraumatic arthritis and its sequelae. Treatment goals in the elderly and low demand patients may be different from those in the general population, focused more on facilitating a situation in which full weight-bearing and preservation of functional autonomy, rather than prevention of posttraumatic arthritis. The natural evolution of posttraumatic osteoarthritis after ankle fracture is dependent on the fracture reduction, stability of the tibiotalar joint, fracture mechanism, initial cartilage lesions, and possibly the role of hindfoot alignment [6]. A systematic review by Stufkens et al. showed that 79.3% of optimally reduced fractures have a good-to-excellent long-term outcome [6].

Treatment options for ankle fractures in general consist of operative and nonoperative treatment. Nonoperative treatment consists of closed reduction and casting (plaster of Paris, synthetic) or close contact casting (CCC). There is a wide array of options for operative management of ankle fractures. Options are non-locking plates, locking plates, titanium elastic fibular nails, locking fibular nails, tension band wiring, external fixation, and retrograde tibiotalar-calcaneal nails. Both operative and nonoperative techniques are associated with a high rate of complications in elderly. Nonoperative treatment of unstable ankle fractures is associated with a nonunion rate of between 48 [7] and 73% [8–10] compared to 0 [11] and 19% [12] after operative treatment. Nonoperative treatment is associated with an increased risk of loss of reduction and subsequent posttraumatic arthritis. Surgically treated ankle fractures are reported to have wound complications in 9 [11] to 23% [12] of cases. Zaghoul et al. found an overall rate of complications in surgically treated ankle fractures in patients over the age of 60 years to be 21.5% with 10.8% of them being major complications prompting surgical intervention for wound washout, removal of implants, and revision of fixation. Smoking, age, diabetes, and local factors (osteopenia, peripheral neuropathy, peripheral vascular disease, lymphedema, and venous insufficiency) were significantly associated with occurrence of complications [13•]. An audit performed by Kurar showed considerably improved anatomical reduction rates following internal fixation but did not report on functional outcomes and patient satisfaction [14].

A recent meta-analysis by Donken et al. was unable to draw conclusions about optimal treatment for ankle fractures [15]. The study included only four eligible trials with a total of 292 patients. All four trials compared open reduction and internal fixation (ORIF) versus closed reduction and plaster cast immobilization. Meta-analyses of functional outcome and pain were not possible due to the incompatible indications for surgery and variations in outcome measures in the included trials. Surgical indications, operative techniques, postsurgical treatment regimens, and conservative treatment were all different. Therefore, the authors conclude that there are insufficient data available to determine whether surgical or conservative treatment produces superior long-term outcomes for ankle fractures [15].

### Treatment in Geriatric Patients

Osteoporotic and neuropathic ankle fractures create an additional challenge in treatment due to the fact that elderly patients frequently have comorbidities such as diabetes, poor wound healing, obesity, peripheral arterial occlusive disease, corticosteroid use, diminished stamina, diminished strength, inability to limit weight-bearing, and/or poor nutritional status. All of these factors are influential in the outcomes of treatment.

Basques et al. found a 5% adverse event after surgery of the fractured ankle in a population of 4412 patients with a mean age of 51 ± 18. An infection rate of 1.7% was found. For both of these, IDDM was associated with an increased rate after ankle fracture ORIF, whereas non-insulin-dependent diabetes mellitus was not. Other associated factors were age > 60 years, American Society of Anesthesiologists (ASA) classification >3, bimalleolar fracture, hypertension, and dependent functional status. Increased ASA class was associated with readmission. A total readmission rate of 3.2% was found [16]. These results are supported by Dodd et al. and Varenne et al. who found the risk factors driving postoperative complications to be an increasing age (respectively >65 and >80 years), obesity, diabetes, ASA score > 2, and functional status [17, 18].

Despite these risks, a recent study by Hsu et al. supports a more aggressive and less expectant management. They found that older patients with ankle fractures were healthier and had a significantly lower 1-year mortality risk than patients treated for a hip fracture or any other diagnosis [19]. However, Smeets et al. found that the costs of surgical treatment of the fractured ankle are double if the patient is over 65 years, compared to younger patients. This was mainly due to a longer total and preoperative stay in the hospital [20].

### Nonoperative Treatment

Only one recent randomized controlled trail (RCT) on the subject was identified. Willet et al. [21•] conducted a pragmatic randomized controlled clinical trial comparing close contact casting (CCC) with open reduction internal fixation (ORIF) for unstable malleolar fractures in patients over 60 years. They compared functional and clinical outcomes. Patients in both study arms were kept on non- or limited weight-bearing status at the discretion of the treating physician. All patients in this study with insulin-dependent diabetes mellitus (IDDM) were excluded [21•]. Six-hundred twenty patients were included, and after 6 months, the Olerud-Molander ankle score (OMAS), post-fracture symptoms, quality of life, pain, ankle motion, patient satisfaction, and mobility were equivalent in both groups. Infection and wound breakdown were more common with surgery. However, it is unclear whether the results of this study will be able to be extrapolated to the group of patients with osteoporotic and neuropathic ankle fractures since the latter were most likely all excluded from this study and rehabilitation without weight-bearing might not be realistic.

### Locking Plates Versus Non-Locking Plates

Locking constructs are widely used to treat osteoporotic fractures. These plates create a fixed angle construct which increases the pull-out strength and decreases the chance of implant failure and secondary loss of reduction. In recent years, biomechanical testing and cadaveric studies have been performed to establish whether the locking plate is in fact superior to the one-third tubular plate which is commonly used.

Bariteau et al. investigated if a combination locked plate with additional fixation options was biomechanically superior in osteoporotic bone and comminuted fracture models [22]. By using an osteoporotic and a comminuted Sawbones model, the fractures were fixed with a lag screw for simple oblique fibula fractures and either a one-third tubular neutralization plate or a locking plate. There was no statistically significant difference in biomechanical testing for simple fractures treated with a lag screw and plate. For comminuted fractures, locked plating demonstrated statistically significant stiffer fixation [22].

Zahn et al. compared a lateral conventional contoured plate with a locking contoured plate stabilizing experimentally induced distal fibular fractures in human cadavers from elderly.

Bone mineral density (BMD) was measured by quantitative computed tomography to correlate the parameters of the biomechanical experiments with bone quality.

They found a higher torque to failure, angle at failure, and maximal torque of the locking plate compared to the conventional plate. In contrast to the non-locking system, fixation with the locking plate was independent of BMD [23].

Recently, Dingemans et al. [24•] performed a meta-analysis of biomechanical studies on reinforced fixation of distal fibular fractures. The two biomechanical outcome measures were torsional stiffness and torque to failure. A total of 13 studies were identified. Six compared locked lateral plating with conventional lateral plating. They could not show a statistically significant difference between the locking and non-locking lateral plates for torque to failure or torsional stiffness. However, locked plating strength was independent from bone mineral density in four studies and, therefore, could make this technique more suitable in the fixation of severe osteoporotic fractures [24•].

In the choice of implant fracture pattern, bone mineralization, costs, and size of the implant should be taken into account. In simple oblique fractures, there is no biomechanical advantage of the locking plate. The locking constructs are often more bulky which can lead to problems during closure and wound breakdown. Also, locking constructs are more expensive to use. Therefore, it is advised to use one-third tubular plates and lag screws in most ankle fractures. The added value of a locking plate may be present for comminuted fractures and those in severely osteoporotic bone.

### Fibular (Locking) Nails

A study by Rajeev et al. assessed the functional outcome of a cohort of patients with fragility fractures of the ankle who were treated with a fibular locking nail. A retrospective review of 24 patients showed a mean period to fracture union of 8.7 weeks. No wound breakdown or any deep infections were reported. All patients were given a lightweight, below-the-knee resting plaster cast and were allowed partial weight-bearing for 6 weeks. After 6 weeks, the plaster cast was removed and all patients were given a walking boot for another 4 weeks. The authors concluded that the use of fibular locking nails to treat these fractures are crucial to achieve early mobilization and also to maintain a good fracture position [25]. One of the interesting things about this study is the immediate partial weight-bearing that was allowed instead of the usual non-weight-bearing. In the elderly population, this may be of great importance.

Understanding the fracture pattern is important in the choice of implant. If there is a syndesmotic injury with lateral displacement of the talus, make sure an intramedullary implant is used that allows for placement of a syndesmotic screw.

### Retrograde Tibiotalar-Calcaneal Nails

A calcaneotalotibial nail can be used in treating an unstable fracture of the ankle in the frail elderly patient. It allows the patient to mobilize immediately. Al Namari et al. studied 48 frail elderly patients with displaced ankle fractures who were treated with a long calcaneotalotibial nail [26•]. The mean age of the group was 82 years (61 to 96) and 85% were females. All were frail, with multiple medical comorbidities, and a mean ASA score of 3 or 4. None could walk independently before their operation. Ninety percent returned to their pre-injury level of function. Complications included superficial infection (4%), deep infection (2%), a broken or loose distal locking screw (6%), valgus malunion (4%), and one below-knee amputation following an unsuccessful vascular operation. There were no cases of nonunion, nail breakage, or peri-prosthetic fracture [26•]. Jonas et al. studied 31 patients with a short retrograde tibiotalar-calcaneal nail and found no postoperative wound problems [27]. They observed three peri-prosthetic fractures possibly due to the fact that short nails were used [27]. The use of a long nail, which crosses the isthmus of the tibia, avoids the risk of peri-prosthetic fractures. In low-demand elderly patients who are not able to partially bear weight and need to maintain their functional autonomy, a retrograde tibiotalar-calcaneal nail might be a viable option.

## Augmentation

Screw stripping is a complicating factor during surgery of the fractured ankle, especially in the osteoporotic bone. Screw stripping can reduce the pullout strength of the screw by more than 80% [28]. Pechon et al. investigated if the pullout strength of the stripped screw holes in the osteoporotic bone could be increased with readily available materials in a cadaveric model. They used stainless steel wire, polysorb suture, or polyethylene terephthalate glycol plastic sheet. All three resulted in a pullout strength that was significantly greater than that of the unaugmented screw, but it was still below than that of the intact construct. It is unknown whether the augmentation would affect the healing process or whether cyclic loading during rehabilitation would cause the augmentation to become dislodged and migrate [29]. Augmentation of screws in the osteoporotic bone with polymethyl methacrylate (PMMA) is commonly used in fractures of the spine [30, 31] and has been reported in calcaneal fractures [32]. There are no reports on the use of PMMA-augmented screws in osteoporotic ankle fractures. Future research and development of augmentation techniques may be relevant in the fractured osteoporotic and neuropathic ankle fracture.

Preliminary Recommendations for Surgical Treatment of Ankle Fractures

**Fig. 1:**
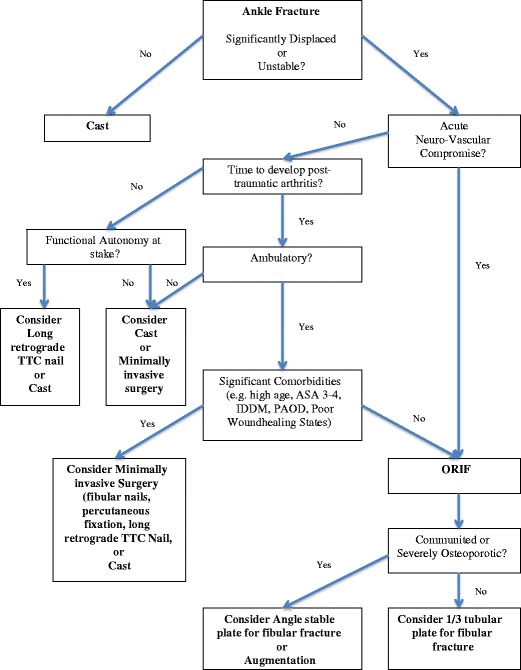
Algorithm decision-making in osteoporotic and neuropathic ankle fractures in the elderly

Even though there is no sufficient evidence to propose specific recommendations for surgery in the elderly patient with an osteoporotic or neuropathic ankle fracture, we propose a general treatment algorithm based on the results of this review (see Fig. 1). When the fractured ankle is considered minimally displaced or stable, the treatment should consist of casting. If the is no fracture, there are indicators such as acute neuro-vascular compromise, time-to-develop posttraumatic arthritis, functional autonomy, pre-traumatic functional status, and comorbidities that will influence the decision on how to treat the fractured ankle in the geriatric population.

## Prevention

An important part of treating ankle fractures in the elderly is prevention. Since osteoporosis is a major contributor to the occurrence of fractures, it is should be prevented or treated early on. The occurrence of a fracture in postmenopausal women and men over 50 approximately doubles the chance of a second fracture [33].

Fracture risk can be assessed using the FRAX-tool which includes age, sex, BMI, previous fractures, family history, smoking, rheumatoid arthritis, alcohol use, and bone mineral density (BMD) [34]. This model does not take the risk of falling into account, which can lead to an underestimation of the actual risk. To incorporate this risk, the Garvan Fracture Risk Calculator can be used [35].

It is recommended to perform a DXA scan in women aged 65 and older and men aged 70 and older, in postmenopausal women and men above the age of 50 based on the risk factor profile, and in postmenopausal women and men aged 50 and older who have had an adult age fracture, to diagnose and determine degree of osteoporosis [33]. Treatment of osteoporosis consists of lifestyle changes like weight loss and alcohol and smoking cessation [33]. Strength and balance exercises can reduce the risk of falling in the elderly [36]. Exclusion of secondary osteoporosis as a cause is important before commencing medical treatment. Medical treatment consists of vitamin D and calcium possibly in combination with bisphosphonates, teriparatide, and monoclonal antibodies such as denosumab [33].

## Conclusion

There is no universal treatment protocol for the elderly patient with a fractured ankle. It is important for the treating physician to not only focus on treating the fractured ankle but on the patient as a whole, to consider comorbidities, medication, prevention, and treatment goals. A multidisciplinary approach, similar to current treatment practices of proximal femur fractures [37], may be advantageous for this particular group of patients. Further research on this topic is necessary to optimize treatment.
